# Importance–Performance Analysis (IPA) of Foodservice Operation, Dietary Life Education, and Nutrition Counseling Tasks of Nutrition Teachers and Dietitians in Jeju, Korea

**DOI:** 10.3390/nu9101157

**Published:** 2017-10-22

**Authors:** Eun A Park, In Sook Chae, Mi Na Jo

**Affiliations:** 1 Department of Nutrition Education, Graduate School of Education, Jeju National University, Jeju 63243, Korea; qkrdmsdk48@naver.com; 2 Department of Food Science and Nutrition, Jeju National University, Jeju 63243, Korea; 3 Division of Hotel and Tourism, The University of Suwon, 17 Wauan-Gil, Hwaseong City, Gyeonggi 18323, Korea

**Keywords:** importance-performance analysis, foodservice operation, dietary life education, nutrition counseling, nutrition teachers, dietitians

## Abstract

The purpose of this study was to analyze foodservice operation, dietary life education, and nutrition counseling tasks of nutrition teachers and dietitians in elementary, middle, and high schools in Jeju, Korea, and to provide effective ways to implement dietary life education and nutrition counseling in schools. This study surveyed 94 nutrition teachers and 46 dietitians working at elementary, middle, and high schools in Jeju during 7–14 May 2015. The importance and performance of 16 tasks of nutrition teachers and dietitians were measured using questionnaires. The data was analyzed by using the SPSS software and Importance–Performance Analysis (IPA). Importance was ranked in the order of foodservice operation (4.72), dietary life education (4.37), and nutrition counseling (4.24); and performance was ranked in the order of foodservice operation (4.48), dietary life education (3.70), and nutrition counseling (3.22). The importance–performance matrix showed that in Quadrant 4, the “Concentrate Here” item was “nutrition and dietary life education for students”, while in Quadrant 2, the “Possible Overkill” item was “cost control and office management”. These findings suggest that it is important to reduce unnecessary administrative and office management tasks in order for nutrition teachers and dietitians to implement effective nutrition education, dietary life education, and nutrition counseling programs.

## 1. Introduction

Jeju Island is located in the south part of Korea and it is the largest island in Korea [1]. The Jeju Special Self-governing Provincial Office of Education decided to focus on school meal nutrition management and the strengthening of dietary life education as priority issues to manage. The “Operational Plan for School Education” includes items related to the safety and nutrition of children’s favorite foods, elimination of unhealthy foods, food safety, and nutrition and dietary life education.

It is important to develop good dietary habits during childhood and adolescence because dietary habits affect growth and are difficult to change. Nutrition and dietary life education advances the nutritional knowledge and dietary behaviors of children and positively affects their overall nutritional intake [2], whereas nutrition counseling improves the dietary lifestyle through customized nutritional management that considers the individual’s nutritional status, disorders, and health status [3].

In schools, nutrition teachers and dietitians are responsible for not only foodservice operation but also dietary life education and nutrition counseling. The Korean government amended the School Meals Act in 2006 and placed nutrition teachers in all schools from 1 March 2007 to improve the nutritional status of students and to provide comprehensive education to form good dietary habits. There are 11,698 elementary, middle, high, and special schools in Korea. Among them, 10,188 schools have school feeding facilities and there are 4928 nutrition teachers (50.0%) and 4932 dietitians (50.0%). In Jeju, 187 elementary, middle, high, and special schools engage in school meals, with 99 nutrition teachers and 74 dietitians working in 2015 [4].

According to the Enforcement Decree of the School Meals Act (Article 8), the work of the nutrition teachers includes providing guidance for dietary lifestyles and nutrition counseling [5,6]. Hence, the role of nutrition teachers and dietitians expands from simple school meal management to nutrition education and nutrition counseling for students [7,8,9,10,11]. However, dietary life education and nutrition counseling through classes did not work properly and there has only been passive dietary life education, such as handout distribution [12]. This is due to the non-allocation of class time, heavy workload, lack of standardized educational processes and materials, and school management’s lack of appreciation for dietary life education and nutrition counseling [13]. Nutrition teachers and dietitians have lots of work to do to manage the foodservice operation, dietary life education, and nutrition counseling, thus the need to check the priority of nutrition teachers’ and dietitians’ tasks by importance–performance analysis (IPA). Martilla and James [14] first suggested IPA as a tool to develop firms’ management strategies. IPA combines measures of importance and performance of each attribute into a two-dimensional grid. The means of importance and performance scores are used as crossing points, and four quadrants are made. Quadrant 1, “Keep Up the Good Work”, indicates the attributes that are important and are performed well. Quadrant 2, “Possible Overkill”, has the attributes that are less important but are performed well. Quadrant 3, “Low Priority”, indicates the attributes that are less important and are not performed well. Quadrant 4, “Concentrate Here”, has the attributes that are important but are not performed well and require focus to improve performance [15]. IPA is a very useful tool to find which attributes should be improved. In this study, we want to find important tasks to be concentrated on among nutrition teachers’ and dietitians’ tasks by using IPA.

There have been several studies on the status of nutrition education in schools [12,13,16,17,18,19]. However, there appears to be a lack of research on the status and perceptions of dietary life education and nutrition counseling. It is only recently that there have been studies on the status of nutrition education and counseling in elementary schools in Seoul [20], the factors relating to nutrition counseling and education for school nutrition teachers in Gyeonggi [21], and the status and perceptions of dietary life education and nutrition counseling by nutrition teachers in elementary schools in Chungbuk [22]. As mentioned above, the Jeju Special Self-governing Province has a high interest in school meal nutrition management and dietary life education program. However, there is a lack of research on the status of dietary life education and nutrition counseling by nutrition teachers and dietitians in schools in the Jeju region.

The main purpose of this study is to analyze the importance and the performance of nutrition teachers and dietitians’ tasks in Jeju, and to find out the important tasks to manage and suggest a point to concentrate on by using the importance–performance analysis.

## 2. Materials and Methods

### 2.1. Subjects and Period of Study

This study conducted a survey among nutrition teachers and dietitians working in elementary, middle, and high schools in Jeju. In 2015, school meals were offered in 187 elementary, middle, high, and special schools, and 99 nutrition teachers and 74 dietitians worked at schools in Jeju. The questionnaires were distributed to the nutrition teachers and dietitians in Jeju who responded by email that they would participate in the survey. The survey was conducted through face-to-face survey, e-documents, and emails from 7 May 2015 to 14 May 2015. A total of 160 questionnaires were distributed, and 140 were collected (87.5%), which were used in the analysis. The Institutional Review Board approval number is JJNU-IRB-2015-016, and it was granted by the Jeju National University Institutional Review Board.

### 2.2. Contents of the Questionnaire

The questionnaire used for this study was developed by revising and complementing the Basic Direction of School Meals, 2015 [12] and other previous studies [20,21,22,23,24]. The questionnaire was composed of 10 questions on demographic and workplace characteristics, as well as 16 questions on the importance and performance of work tasks of nutrition teachers and dietitians. The scale items for importance-performance analysis were presented in Appendix Table A1.

With regard to demographic characteristics of the research targets, age, number of years at the job, employment type, and level of education were identified. For workplace characteristics, school establishment type, school type, school meal type, school meal operations, number of students eating school meals, and number of meals were identified.

The main jobs of nutrition teachers and dietitians were foodservice operation, nutrition and dietary life education, and nutrition counseling. The eight items on foodservice operation included menu planning, purchase management, cooking and serving, facility management, sanitation management, human resource management, quality management, and cost control and office management. The five items on nutrition and dietary life education included nutrition and dietary life education planning, nutrition and dietary life educational materials development, nutrition and dietary life education for students, nutrition and dietary life education for teachers to guide students, and nutrition and dietary life education for parents to guide students. The three items on nutrition counseling included nutrition counseling for students, nutrition counseling for teachers of students requiring special attention, and nutrition counseling for parents of students requiring special attention.

Each item was scored on a 5-point Likert scale, with the measurement of task importance ranging from 1 (very unimportant) to 5 (very important), and the measurement of task performance ranging from 1 (very dissatisfied) to 5 (very satisfied).

### 2.3. Statistical Analysis

In this study, the final data from 140 questionnaires was analyzed using SPSS 18.0 (SPSS, Inc. Chicago, IL, USA). A paired-sample *t*-test was used to compare the mean of importance and performance data. An independent *t*-test and ANOVA were conducted to observe differences according to demographic and workplace characteristics. Duncan’s multiple-range test was performed as a post hoc test of ANOVA.

To construct the importance–performance matrix, the means of the performance and importance ratings of each attribute were calculated. The attributes in Quadrant 1 are high-importance and high-performance and are labelled “Keep Up the Good Work”. The attributes in Quadrant 2 are low-importance and high-performance and are labelled “Possible Overkill”. The attributes in Quadrant 3 are low-importance and low-performance and they are “Low Priority”. The attributes in Quadrant 4 are high-importance and low-performance and are labelled “Concentrate Here” [14,25].

## 3. Results

### 3.1. Demographic and Workplace Characteristics

The demographic and workplace characteristics are shown in Table 1. The participants were generally aged between 40 and 49 years (57.9%), followed by 30–39 years old (17.1%), over 50 (14.3%), and below 30 (10.7%). The number of years at the job was in the order of from 10 to 20 years (37.9%), below 10 years (35.0%), and more than 20 years (27.1%). Employment type showed that there were more nutrition teachers (67.2%) compared to dietitians (32.8%). More participants had attained their highest education level from a university (67.9%), compared to post-graduate schools (32.1%). The analysis of workplace characteristics indicated that the school establishment type was generally skewed towards national/public (89.3%) rather than private (10.7%). The school types were led by elementary schools (55.0%), followed by middle school (22.1%), high school (17.9%), and then others (5.0%). The type of school meals was skewed towards urban (55.7%) rather than rural and islandic styles (44.3%). School meal operations were led by individual management of meals (90.7%) rather than joint management and cooking (9.3%). The number of students eating school meals was led by the less than 500 group (57.1%), followed by the less than 1000 group (22.9%), and the more than 1000 group (20.0%). In terms of number of meals, the 1 meal per day (84.3%) selection was more than that of 2 or 3 meals per day (15.7%).

### 3.2. Difference between Nutrition Teachers and Dietitians According to Demographic and Workplace Characteristics

An analysis of the demographic characteristics and differences in the work environment among nutrition teachers and dietitians indicated that there were statistically significant differences according to age (*p* < 0.01), job experience (*p* < 0.001), level of education (*p* < 0.001), school establishment type (*p* < 0.001) and school meal operation (*p* < 0.01) (Table 2). It was found that nutrition teachers were generally older than dietitians. Nutrition teachers also had longer job experiences than dietitians. However, dietitians were found to have higher levels of education. In terms of school establishment type, nutrition teachers worked generally in national and public schools, while dietitians worked in not only national and public schools but also in private schools. In terms of school meal operation types, nutrition teachers had engaged in individual cooking and in joint management and cooking, while all dietitians had engaged in individual cooking.

### 3.3. Difference between the Importance and Performance of Nutrition Teachers’ and Dietitians’ Tasks

The tasks of the nutrition teachers and dietitians were divided into three categories—foodservice operation, dietary life education, and nutrition—in order to analyze their importance and performance (Table 3). The results of the analysis indicated that the importance ratings of all categories were significantly higher than the performance ratings (*p* < 0.05). Foodservice operation showed the highest importance score, followed by nutrition and dietary life education, then nutrition counseling. Foodservice operation also had the highest performance score, followed by nutrition and dietary life education, then nutrition counseling. In the foodservice operation part, sanitation management was of highest importance, while purchase management had the highest performance. In the dietary life education part, importance was highest for education for students, while nutrition & dietary life education planning had the highest performance. In the nutrition counseling part, the highest importance was ranked in the order of nutrition counseling for students, nutrition counseling for parents of students requiring special attention, then nutrition counseling for teachers of students requiring special attention; on the other hand, highest performance was in the order of nutrition counseling for students, nutrition counseling for teachers of students requiring special attention, and nutrition counseling for parents of students requiring special attention.

### 3.4. Importance–Performance Analysis (IPA) of Nutrition Teachers’ and Dietitians’ Tasks

**Figure 1:**
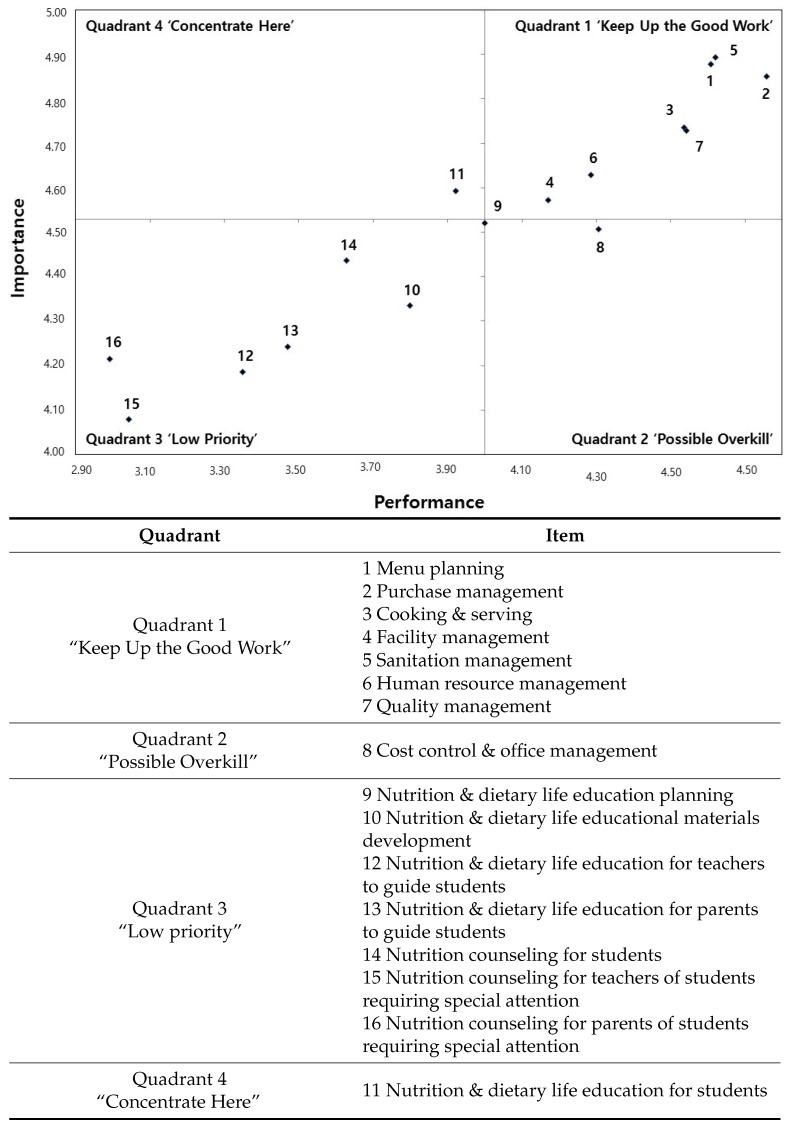
Importance–performance analysis of nutrition teachers’ and dietitians’ tasks.

There were statistically significant differences between performance and importance in all 16 task items for nutrition teachers and dietitians (Table 3). Therefore, there was room for improvement on all items. As such, this study has conducted the Importance–Performance Analysis (IPA) to set long- and short-term operational improvement strategies by identifying priorities depending on the needs of the consumers and presenting them on a grid (Figure 1). They were divided into 4 quadrants with the base of 4.53 importance points and 4.00 performance points on average.

Quadrant 1 can be termed as “Keep Up the Good Work” as it has high importance and performance, wherein the work items are carried out in accordance to the perception of importance by the consumer; this includes (1) menu planning; (2) purchase management; (3) cooking and serving; (4) facility management; (5) sanitation management; (6) human resource management; and (7) quality management. Items in Quadrant 1 generally cater to foodservice operation; their current level of performance should be maintained and continuously improved. According to Table 3, the importance perceived by the consumer and the perceived level of performance were different in all items; hence, methods to decrease the gap between performance and importance should continuously be sought, even though this is the quadrant where maintaining present levels is sufficient.

Quadrant 2 has a high level of performance despite a low level of importance and can be termed “Possible Overkill”. This quadrant includes (8) cost control and office management, and relates to administrative tasks in meal management that are carried out more frequently than is required for their level of importance.

Quadrant 3 is a zone of “Low Priority” with low importance and low performance, and includes (9) nutrition and dietary life education planning; (10) nutrition and dietary life educational materials development; (12) nutrition and dietary life education for teachers to guide students; (13) nutrition and dietary life education for parents to guide students; (14) nutrition counseling for students; (15) nutrition counseling for teachers of students requiring special attention; and (16) nutrition counseling for parents of students requiring special attention. Items relating to Quadrant 3 generally relate to nutrition and dietary life education and nutrition counseling, and were not perceived as important as tasks relating to foodservice operation in Jeju, Korea.

Quadrant 4 is termed “Concentrate Here” with high importance but comparatively low performance, resulting in dissatisfied consumers; items belonging here need to be prioritized in the short term. This quadrant includes (11) nutrition and dietary life education for students, and this item requires focus and execution in the near future.

## 4. Discussion

This study examined the tasks of nutrition teachers and dietitians in elementary, middle, and high schools in the three categories of foodservice operation, nutrition and dietary life education, and nutrition counseling. Their importance and performance were also analyzed to identify key areas requiring management among the tasks of nutrition teachers in Jeju. Through these steps, this study aimed to identify methodologies for comprehensive and practical dietary life education and nutrition counseling.

Nutrition teachers were generally found to be older than dietitians, and with longer work experience, although dietitians had a higher level of education. Han [11] also stated that nutrition teachers were generally older than dietitians. In terms of school establishment type, nutrition teachers generally worked in national and public schools, whereas dietitians worked in national, public, and private schools. In terms of foodservice operations, nutrition teachers generally cooked individually with some joint management and cooking, while dietitians only cooked individually.

Under the Enforcement Decree of the Elementary and Secondary Education Act amended in 2003, when a school cannot hire a nutrition teacher due to reasons such as a high demand for teachers, the school must hire a licensed dietitian [26,27]. Following the School Meals Act amended in 2006, a school dietitian is considered a public official for hygiene from 2007, and is generally changed to a nutrition teacher upon passing the teacher certification exams. While nutrition teachers are full-time school nutritionists, dietitians often share the same work as nutrition teachers but are not treated like them and tend to be on contract [8,27].

Moreover, a study indicated that there were more than twice the number of dietitians with degrees higher than Master’s compared to nutrition teachers, which was in line with the findings of this study. The problem was that dietitians with no education degree had gone to graduate schools, which certified them as nutrition teachers; however, there were very few new-hires for nutrition teachers.

In analyzing the importance and performance of the work of nutrition teachers and dietitians, divided into the three categories of foodservice operation, dietary life education, and nutrition counseling, the importance of all three categories was significantly higher than their performance. Moreover, compared to the tasks relating to foodservice operation, the importance and performance of dietary life education and nutrition counseling were ranked lower. In a study on nutrition teachers in Seoul by Park [20], tasks relating to nutrition education and counseling had lower performance and perceptions of importance compared to foodservice operations, and this was also in line with the results of this study.

The results of the IPA have included mainly foodservice operation items in Quadrant 1, “Keep up the Good Work”. Their current level of performance should be maintained and continuously improved. Quadrant 2, “Possible Overkill”, included “cost and administrative management,” and the indicated that this item was being carried out more frequently than is required for its level of perceived importance. Yang [28] and You et al. [29] also indicated that the administrative tasks of nutrition teachers and dietitians were excessive, which are in line with this study. According to Yang [28], among the tasks that require job improvement and reduction, “simplification of administrative documents” was highest at 37.9%, “reducing official documents on school meals” was at 25.0%, and “transferring other tasks” was at 19.7%, revealing a high demand for reducing administrative work. Regarding tasks with work transfer and reduction, You et al. [29] indicated high response rates of 109 (35.1%) for “support for school meal costs and milk”, 83 (26.7%) for “reporting and reconciling budgets”, and 75 (24.1%) for “various requested and reporting documents”. It was clear that nutrition teachers were concerned and stressed with overwork and wanted work transfer and reduction to effectively manage it. Thus, it is important to reduce cost and administrative work such as office management, and strengthen the original work function of nutrition teachers and dietitians.

Items in Quadrant 3 generally relate to nutrition and dietary life education and nutrition counseling, and were not perceived as important as tasks relating to foodservice operation in Jeju. Han [11] also indicated that nutrition counseling had low work performance and was perceived as low importance by nutrition teachers and dietitians, regardless of rank. Such results indicate that school nutrition teachers and dietitians perceive the setting of nutrition education plans, nutrition education execution, and nutrition counseling to be important but difficult; difficulties associated with nutrition education and counseling due to low levels of knowledge and confidence and low perceptions of school management were in line with other studies [17,30,31,32,33]. Hence, there must be institutional support and education for school nutritionists to become proficient in nutrition education and counseling.

Quadrant 4 includes “nutrition and dietary life education for students”, and this quadrant requires focused management. While there are legal foundations of placing nutrition teachers in schools, there is weak institutional support to help these nutrition teachers carry out their duties within schools. They are typically unable to engage in their original role of providing nutrition education, and focus instead on the existing foodservice operations. According to existing studies on nutrition education [17,34], more than 95% of principals, parents, and teachers indicated that nutrition education was required, and more than 85% believed that nutrition education should be delivered by nutrition teachers. However, despite the high perceived necessity of nutrition education, the actual level of execution was low. While elementary schools saw more active execution of nutrition education compared to middle and high schools [35,36], the direct delivery of nutrition education in Seoul and Gyeonggi regions was 36.8%, 26.6% in Gangwon Province [37], and less than 10% in Jeonbuk [38].

Kim [39] asserted that the job satisfaction levels of nutrition teachers were lower than the expected levels before they became nutrition teachers. This results from the high expectations from authority and independence on nutrition counseling and education, and its discrepancy with the reality where they are perceived simply as managers of school meals.

You et al. [29] identified the perception and level of necessity on the items that require supplementation relating to the tasks and institutions for nutrition teachers. In school, dietary life education is carried out through Practical Education, Technology and Home, and Physical Education. Moreover, dietary life education by nutrition teachers and dietitians is being carried out through topics such as obesity education, dietary life and cultures, managing picky eating, cooking practicums, experiencing kitchens, meal etiquettes, nutrition, and practicing tradition. However, due to the excessive amount of work expected of nutrition teachers and dietitians, the burden of operating a class, and the lack of independent educational curricula, the dietary life education offered in schools faces various obstacles [40].

Moreover, there are various guidelines such as the guidelines for nutrition and dietary life by the Ministry of Food and Drug Safety, Children’s Dietary Lifestyles Guide [41], and Guidelines for Green Dietary Lifestyle [42]. However, they remain insufficient to meet the diverse level of educational demands and skill levels of students [43]. According to Park et al. [16], 59.5% of dietitians engaged in nutrition education and its methods; however, majority of the education is carried out using handouts and is indirect in nature. Among the reasons for not engaging in nutrition education, excessive amount of work was the most cited (75.8%). After the introduction of nutrition teachers, Jun [19] indicated that 80% of the research targets engaged in indirect nutrition education, and the reasons for not being able to engage in direct education were the lack of educational opportunities (36.9%) and excessive work relating to foodservice operation (33.3%). In a study of the status of nutrition education in the Gyeongnam region [18], the reason for being unable to engage in direct nutrition education was mainly the excessive work relating to foodservice operation (34.2%). In a study focusing on elementary schools nationwide [17], the reasons for the same were ranked in the order of institutional reasons (62.5%) and lack of time due to excessive amounts of work (62%), similar to the results of this study. The study by Yoon [35] indicated that institutional problems must be addressed as nutrition education was generally indirect (72.4%); methods of improving and introducing direct education included a comprehensive education process, diversification of educational programs, and allotment of time for education and perceptions of school management. Kim et al. [22] indicated that to invigorate the dietary life education for elementary schools in Chungbuk, introduction of an independent class for dietary life education was found to be the most important (44.2%). In a study by Jung and Lee [13], the development of learning materials by school year (4.28) and regularity of education (4.26) were presented as potential solutions.

Based on the results of this study, we provide recommendations for achieving practical and comprehensive nutrition and dietary life education and nutrition counseling, whose performance was found to be much lower than foodservice operation. Nutrition teachers and dietitians face difficulties in offering education and counseling on nutrition due to the excessive work relating to school meals and administrative tasks, resulting in a lack of time for the education and counseling. Hence, there must be methods to decrease the unnecessary administrative work so that nutrition and dietary life education and counseling can be executed effectively. Moreover, it should be granted independent class time, settling eventually as a regular class by itself. The development of various educational materials on nutrition and dietary life is also critical to ensure that the classes meet the various educational needs and skill levels of students.

## 5. Conclusions

Among the tasks of nutrition teachers and dietitians, nutrition and dietary life education for students is the most important to concentrate on, while cost control and office management tasks should be reduced. In order for nutrition teachers and dietitians to implement effective nutrition and dietary education, it is important to reduce unnecessary administrative and office management tasks.

## Figures and Tables

**Table 1:** Demographic characteristics and workplace characteristics of subjects.

Category		*N*	%
Age (year)	<30	15	10.7
	30–39	24	17.1
	40–49	81	57.9
	>50	20	14.3
The number of years at the job	<10	49	35.0
	10–20	53	37.9
	>20	38	27.1
Employment type	Nutrition teacher	94	67.2
	Dietitian	46	32.8
Education	Graduated from university	95	67.9
	Post-graduate schools	45	32.1
School establishment type	National/Public School	125	89.3
	Private School	15	10.7
School type	Primary School	77	55.0
	Middle School	31	22.1
	High School	25	17.9
	Others	7	5.0
Type of school meals	Urban	78	55.7
	Rural and islandic	62	44.3
School meal operation	Individual management of meals	127	90.7
	Joint management and cooking	13	9.3
Number of students eating school meals	Less than 500	80	57.1
	Less than 1000	32	22.9
	More than 1000	28	20.0
Number of meals	1 meal/day	118	84.3
	2 or 3 meals/day	22	15.7
Total		140	100.0

**Table 2:** Differences between nutrition teachers and dietitians by demographic characteristics and workplace characteristics.

Category		Nutrition Teachers		Dietitians		*p*-Value
		*N*	%	*N*	%	
Age (year)	<30	11	11.7	4	8.7	0.002
	30–39	9	9.6	15	32.6	
	40–49	56	59.6	25	54.3	
	>50	18	19.1	2	4.3	
The number of years at the job	<10	29	30.9	20	43.5	<0.001
	10–20	29	30.9	24	52.2	
	>20	36	38.3	2	4.3	
Education	Graduated from university	75	79.8	20	43.5	<0.001
	Post-graduate schools	19	20.2	26	56.5	
School establishment type	National/Public School	92	97.9	33	71.7	<0.001
	Private School	2	2.1	13	28.3	
School type	Primary School	53	56.4	24	52.2	0.886
	Middle School	19	20.2	12	26.1	
	High School	17	18.1	8	17.4	
	Others	5	5.3	2	4.3	
Type of school meals	Urban	48	51.1	30	65.2	0.113
	Rural and islandic	46	48.9	16	34.8	
School meal operation	Individual management of meals	81	86.2	46	100.0	0.008
	Joint management and cooking	13	13.8	0	0.0	
Number of students eating school meals	Less than 500	51	54.3	29	63.0	0.614
	Less than 1000	23	24.5	9	19.6	
	More than 1000	20	21.3	8	17.4	
Number of meals	1 meal/day	81	86.2	37	80.4	0.381
	2 or 3 meals/day	13	13.8	9	19.6	

**Table 3:** Comparative analysis of importance and performance of nutrition teachers’ and dietitians’ job.

Category	Item	Importance ^(1)^	Performance ^(2)^	*p*-Value
Foodservice operation	1. Menu planning	4.88 ± 0.32	4.61 ± 0.63	<0.001
	2. Purchase management	4.85 ± 0.39	4.76 ± 0.47	0.032
	3. Cooking & serving	4.74 ± 0.47	4.54 ± 0.60	<0.001
	4. Facility management	4.57 ± 0.53	4.17 ± 0.74	<0.001
	5. Sanitation management	4.89 ± 0.31	4.62 ± 0.54	<0.001
	6. Human resource management	4.63 ± 0.54	4.29 ± 0.75	<0.001
	7. Quality management	4.73 ± 0.46	4.54 ± 0.61	<0.001
	8. Cost control & office management	4.51 ± 0.61	4.31 ± 0.72	0.001
	Average	4.72 ± 0.33	4.48 ± 0.49	<0.001
Dietary life education	9. Nutrition & dietary life education planning	4.52 ± 0.58	4.00 ± 0.79	<0.001
	10. Nutrition & dietary life educational materials development	4.34 ± 0.66	3.80 ± 0.84	<0.001
	11. Nutrition & dietary life education for students	4.59 ± 0.56	3.92 ± 0.82	<0.001
	12. Nutrition & dietary life education for teachers to guide students	4.19 ± 0.78	3.35 ± 0.88	<0.001
	13 Nutrition & dietary life education for parents to guide students	4.24 ± 0.68	3.47 ± 0.80	<0.001
	Average	4.37 ± 0.55	3.70 ± 0.65	<0.001
Nutrition counseling	14. Nutrition counseling for students	4.44 ± 0.64	3.63 ± 0.78	<0.001
	15. Nutrition counseling for teachers of students requiring special attention	4.08 ± 0.81	3.04 ± 0.95	<0.001
	16. Nutrition counseling for parents of students requiring special attention	4.21 ± 0.74	2.99 ± 0.89	<0.001
	Average	4.24 ± 0.64	3.22 ± 0.76	<0.001
Average		4.53 ± 0.39	4.00 ± 0.46	<0.001

^(1)^ 5-point Likert scales 1: very unimportant 5: very important; ^(2)^ 5-point Likert scales 1: very dissatisfied 5: very satisfied.

## Appendix

**Table A1:** Scale items for importance–performance analysis.

Category	Item	Importance					Performance				
Foodservice operation	1 Menu planning	1	2	3	4	5	1	2	3	4	5
	2 Purchase management	1	2	3	4	5	1	2	3	4	5
	3 Cooking & serving	1	2	3	4	5	1	2	3	4	5
	4 Facility management	1	2	3	4	5	1	2	3	4	5
	5 Sanitation management	1	2	3	4	5	1	2	3	4	5
	6 Human resource management	1	2	3	4	5	1	2	3	4	5
	7 Quality management	1	2	3	4	5	1	2	3	4	5
	8 Cost control & office management	1	2	3	4	5	1	2	3	4	5
Dietary education	9 Nutrition & dietary life education planning	1	2	3	4	5	1	2	3	4	5
	10 Nutrition & dietary life educational materials development	1	2	3	4	5	1	2	3	4	5
	11 Nutrition & dietary life education for students	1	2	3	4	5	1	2	3	4	5
	12 Nutrition & dietary life education for teachers to guide students	1	2	3	4	5	1	2	3	4	5
	13 Nutrition & dietary life education for parents to guide students	1	2	3	4	5	1	2	3	4	5
Nutrition Counseling	14 Nutrition counseling for students	1	2	3	4	5	1	2	3	4	5
	15 Nutrition counseling for teachers of students requiring special attention	1	2	3	4	5	1	2	3	4	5
	16 Nutrition counseling for parents of students requiring special attention	1	2	3	4	5	1	2	3	4	5

Importance 1: very unimportant, 5: very important, Performance 1: very dissatisfied, 5: very satisfied.
